# Dealing with Misfolded Proteins: Examining the Neuroprotective Role of Molecular Chaperones in Neurodegeneration 

**DOI:** 10.3390/molecules15106859

**Published:** 2010-10-08

**Authors:** Yousuf O. Ali, Brandon M. Kitay, R. Grace Zhai

**Affiliations:** 1 Department of Molecular and Cellular Pharmacology, University of Miami, Miller School of Medicine, Miami, FL 33136, USA; 2 Neuroscience Graduate Program, University of Miami, Miller School of Medicine, Miami, FL 33136, USA

**Keywords:** Alzheimer’s disease, Parkinson’s disease, PolyQ disease, Hsp90, Hsp70

## Abstract

Human neurodegenerative diseases arise from a wide array of genetic and environmental factors. Despite the diversity in etiology, many of these diseases are considered "conformational" in nature, characterized by the accumulation of pathological, misfolded proteins. These misfolded proteins can induce cellular stress by overloading the proteolytic machinery, ultimately resulting in the accumulation and deposition of aggregated protein species that are cytotoxic. Misfolded proteins may also form aberrant, non-physiological protein-protein interactions leading to the sequestration of other normal proteins essential for cellular functions. The progression of such disease may therefore be viewed as a failure of normal protein homeostasis, a process that involves a network of molecules regulating the synthesis, folding, translocation and clearance of proteins. Molecular chaperones are highly conserved proteins involved in the folding of nascent proteins, and the repair of proteins that have lost their typical conformations. These functions have therefore made molecular chaperones an active area of investigation within the field of conformational diseases. This review will discuss the role of molecular chaperones in neurodegenerative diseases, highlighting their functional classification, regulation, and therapeutic potential for such diseases.

## 1. Introduction

A significant percentage of the aging population suffers from neurodegenerative conditions, which are among the most intractable of diseases. Decades of both clinical and basic science research have discovered and characterized dozens of neurodegenerative disorders, triggered by a variety of genetic and environmental factors. Compared to other cell types, neurons are especially susceptible to degeneration because of their long lifetime (the entire lifespan of the organism for most neurons), and, when damaged or lost, they are not readily replenished through cell division, with the exception of neurons in special neurogenic zones in vertebrates [1]. The long life of neurons demands a high level of neuronal maintenance and protection. Healthy neurons are able to maintain their integrity throughout the lifespan, suggesting the existence of a maintenance mechanism that allows neurons to sustain or even repair damage. Chaperones are the main workforce for cellular maintenance and stress response. Research on neurodegenerative diseases in recent years has uncovered the tremendous neuroprotective properties of chaperones and has since placed chaperones in the center stage of neuroprotection. Hence, stimulating and augmenting the intrinsic chaperone activity in the nervous system has become a main focus in the design of many neuroprotective strategies. 

In this review, we first provide an overview of the different types of chaperones in the nervous system. Then we highlight the mechanisms of chaperone regulation with the focus on augmenting the protective activity of chaperones. Next we review in detail the role of chaperones in several neurodegenerative conditions, including Parkinson’s disease (PD), Alzheimer’s disease (AD), Polyglutamin diseases (PolyQ disease), as well as ischemia and stroke. We also discuss several known diseases that are caused by mutations in chaperones. Finally, we review the pharmacological modulators of molecular chaperones that either have been used or have the potential to be used as therapeutics for neurodegenerative diseases. 

## 2. Functional Classification of Chaperones

Molecular chaperones are a family of proteins that facilitate and regulate proper protein folding. In other words, they bind to and stabilize proper conformation of client proteins, and, through cycles of regulated binding and release, facilitate their correct fate by preventing inappropriate misfolding [2]. Based on their abundance at any one time in a cell, chaperones can be categorized into three groups: 1) constitutively expressed; 2) constitutively expressed and induced (upon stress, Hsp90); and 3) only inducible (upon stress, Hsp70) [3]. 

### 2.1. Constitutively expressed

Although the information detailing how a native protein should fold is encoded in a protein’s linear sequence of amino acids [4], the crowded microenvironment of the cellular milieu fosters intermolecular interactions that promote aggregation of newly synthesized proteins rather than productive protein folding (referred to as the excluded volume effect) [5,6]. Constitutively expressed molecular chaperones primarily serve to shield newly synthesized proteins from such interactions, generally by interacting with stretches of hydrophobic residues or polypeptide chains lacking secondary structure, assisting in the maintenance of protein solubility, and promoting proper folding [5]. The subcellular location at which a protein is synthesized is typically dictated by a protein’s final destination, and specialized networks of constitutively expressed chaperones exist to aid in folding proteins in distinct cellular compartments. 

#### 2.1.1. Constitutively expressed cytosolic chaperones

A broad range of client proteins in the eukaryotic cytosol are chaperoned co-translationally by the 70-kDa heat shock protein cognate protein (Hsc70) of the heat shock protein 70 (Hsp70) family of proteins [7,8,9]. Hsc70 is comprised of a ~44-kDa N-terminal ATPase domain and a ~27-kDa C-terminal peptide binding domain [10]. In conjunction with its J-domain containing co-chaperone HDJ1 (or HDJ2; also referred to as 40-kDa heat shock protein (Hsp40)), Hsc70 facilitates the folding of large [11], often multi-domain proteins greater than 50-kDa [12] in an ATP-dependent manner [5]. Hsc70 often functions together with the 90-kDa heat shock protein (Hsp90) and may be considered a single, multi-chaperone complex mediating the folding of a smaller subset of client proteins that includes steroid hormone receptors [13,14], regulatory kinases, and transcription factors [15,16]. It is also interesting to note that by complexing with a large host of co-chaperone proteins, Hsc70/Hsp90 are capable of additional functions including uncoating of clatherin coated vesicles, protein translocation, and cytoskeleton assembly [17]. The 60-kDa heat shock protein (Hsp60) family proteins (also referred to as chaperonins) are double-ring shaped heptameric protein complexes forming a large central cavity within which protein folding takes place [10]. The TCP-1 ring complex (TRiC, also referred to as CT for chaperonin-containing CCT) is a Group II chaperonin that also contributes to the folding of proteins in the eukaryotic cytosol [5]. TRiC has been shown to be critical for the folding of the cytoskeletal proteins β-actin [18] and tubulin [19] in addition to other specific protein clients including Gα-transducin subunit, von Hippel-Lindau (VHL) tumor suppressor, anaphase promoting complex (APC), and cyclin E [20,21,22,23]. 

#### 2.1.2. Constitutively expressed ER chaperones

Proteins destined for incorporation into membranes or the secretory pathway are synthesized and transported co-translationally into the lumen of the endoplasmic reticulum (ER) [24,25]. Studded with ribosomes, the ER is a highly active site of protein synthesis through which nearly 1/3 of the eukaryotic proteome is synthesized as unfolded polypeptides [26]. A large proportion of the protein content of the ER is therefore dedicated to assisting in protein folding, so much so that chaperones far outnumber their newly synthesized client proteins in the ER lumen [27]. This extensive network of ER chaperones additionally establishes a stringent mechanism of protein quality control (QC) by funneling proteins incapable of appropriately folding through a highly regulated ER-associated degradation pathway (ERAD) [28,29]; as proteins progressing along the secretory pathway lack access to folding support, it is essential that every protein assume its native conformation prior to embarking from the ER [24,30]. The most abundant resident ER chaperone in mammals is the Hsp70 family related immunoglobulin heavy chain binding protein (BiP, also referred to as 78-kDa glucose regulated protein, Grp78), which can immediately interact and aid with drawing newly synthesized proteins through the ER translocation pore, the translocon, as BiP has been demonstrated to form a gating cap on the luminal side of the transolocon [27,31,32]. BiP contains an N-terminal ATPase domain which, through ATP hydrolysis and ADP exchange, regulates client binding and release at its C-terminal protein substrate binding domain [33]. A number of Hsp40 family J-domain containing proteins have been found in mammals (ER-localized DnaJ homologues, ERdj1-5) that act as BiP co-chaperones by facilitating nucleotide hydrolysis and exchange [10,34,35]. The ER resident Hsp90 family chaperone 94-kDa glucose regulated protein (Grp94) is also highly expressed in the ER and, similar to that of Hsc70 and Hsp90 in the cytosol, facilitates the folding of client proteins that it sequentially receives from BiP, such as immunoglobulins [36] and procollagen [37]. Unique to the ER are chaperones that interact exclusively with secretory and membrane proteins covalently modified by N-linked glycosylation of oligosaccharides (glycoproteins) [38]. The ER transmembrane chaperone calnexin, and its soluble homologue calreticulin, are lectin chaperones that recognize proteins retaining one or more monoglucosylated N-glycan sidechains [38], promoting folding in an ATP-independent manner [39]. The lectin chaperones most readily contribute to protein folding through their complexing with ERp57, a protein disulfide isomerase (PDI) family member [40]. Through two thioredoxin motifs, ERp57 has thiol-dependent reductase activity that catalyzes the formation of nascent disulphide bonds specifically in N-glycoproteins [38]. The complex of calnexin/calreticulin with ERp57 forms a unique cage-like structure, similar to that of Hsp60 in the cytosol [24]. The presence of the lectin chaperones has also been shown to enhance the disulfide isomerase activity of ERp57 [41].

#### 2.1.3. Constitutively expressed “moonlighting” chaperones

Other constitutive chaperones have been described serving mitochondria-specific protein clients [42,43] as well as clients specific to nuclear roles such as histones during DNA replication and repair [44]. A new class of “nontraditional” constitutively expressed chaperones is also emerging as a number of proteins, often referred to as proteins that “moonlight”, are being discovered to multitask, performing multiple unrelated functions [45,46,47]. For example, peroxiredoxin (Prx) is a thioredoxin-dependent antioxidant protein that also retains chaperone activity [48]. This constitutively expressed protein exists in its low molecular weight form to catabolize reactive oxygen species (ROS) such as peroxide, peroxinitrite, and other organic hydryoperoxides, but may oligomerize into high molecular weight chaperone complexes upon both oxidative and heat shock stress [48]. This dual peroxidase-chaperone function has been demonstrated for both yeast [48] and human [49] homologues of the Prx protein, and can be differentially regulated by site-specific phosphorylation [50]. Nicotinamide mononucleotide adenylyl transferase (NMNAT) is a constitutively expressed protein and key enzyme in the reaction that catalyzes the condensation of nicotinamide mononucleotide (NMN) and adenosine triphosphate (ATP) to nicotinamide adenine dinucleotide (NAD+), a coenzyme critical to metabolic redox reactions of the cell [51,52]. In addition to its NAD+ synthase activity, NMNAT has also been demonstrated to have chaperone activity that can afford neuroprotection in response to neuronal proteotoxic stress, independent of its enzymatic activity [53,54]. Collectively, this new class of constitutively expressed, moonlighting chaperones may serve as an immediate, first responder to various forms of stress prior to mounting an induced chaperone response.

### 2.2. Constitutively expressed and induced: Hsp90

Hsp90 is present in most cellular compartments such as the cytosol, endoplasmic reticulum (ER), mitochondria (and chloroplast in plants), and is one of the most abundant proteins in eukaryotic cells, comprising 1–2% of total proteins under unstressed conditions [55]. Hsp90 is indispensible for cell survival, playing an important role in the folding of at least 200 specific proteins in essential signaling pathways, and in the refolding of denatured proteins after stress [56,57]. Hsp90 is an evolutionarily conserved ATP-dependent chaperone, with a very unique N-terminal ATP binding site (in addition to another C-terminal nucleotide binding site) that has allowed for the development of very specific Hsp90 inhibitors such as geldanamycin, a macrocyclic antitumor agent [58]. 

Hsp90 is quite unique from other heat shock chaperones: it does not bind to non-native proteins but rather to substrates in their native states and targets a specific set of client proteins that are involved in signal transduction [59]. Hsp90 has been shown to interact with important kinases that are known to function as hubs integrating multiple inputs [60]. These multifunctional client kinases include ErbB2, Src, Abl or Met tyrosine kinases, and cyclin-dependent serine kinases [57], which are a part of an intricate multidimensional signaling web that integrate information from various levels. The functional consequence of Hsp90 interaction with these kinases is possibly to regulate the specificity of activation of these signaling pathways by controlling the potential of its clients to interact with each other [61]. In fact, many of these client proteins are known to be bound to Hsp90 in an inactive state and are activated upon dissociation from Hsp90. For example, the catalytic domain of raf proteins, members of MAP kinase kinase kinases (MEKKs), are in a heterocomplex with Hsp90, and are activated upon dissociation from Hsp90 such as in stress conditions [62]. Moreover, the abundance of Hsp90 in an unstressed cell enables it to bind to and keep stress transcription factors such as Heat Shock Factor-1 (HSF-1) in a monomeric inert state [56]. Upon heat shock, HSF-1 is relieved from Hsp90 complex due to a surge in demand for chaperones to protect from protein misfolding. This is the first step in the activation of HSF-1 (discussed later in section 3.1). 

Hsp90 is constitutively expressed, but heat shock also causes induction of Hsp90 [63]. In the cytoplasm of *Saccharomyces cerevisiae* (a yeast), there are two isoforms of Hsp90: the heat shock induced Hsp82, and the constitutively expressed Hsc82 [64]. The inducible Hsp90 offers a negative feedback loop to control the transcriptional activity of HSF-1 [63].

### 2.3. Inducible: Hsp70

The Hsp70 family has several members, some of which are stress-inducible (Hsp70, Hsp70i), while others are constitutively expressed (Hsc70). The inducible Hsp70 proteins are among the first to be up-regulated upon heat shock to cope with the immediate protein misfolding stress. Hsp70 chaperones are found in most cellular compartments, including the nucleus and cytoplasm (Hsc70), mitochondria (mortallin), and ER (Grp78) [65]. Hsp70 proteins have two unique domains critical for their chaperone function: an N-terminal ATPase domain and a C-terminal substrate binding domain [65]. ATP hydrolysis in the N-terminal domain causes a conformational change in the client binding domain, which is composed of a base of beta strands and a lid which closes upon ATP hydrolysis to form a clamp [66]. The clamp structure allows the binding to short extended hydrophobic regions of the misfolded client proteins and therefore prevents aggregation of the misfolded clients [66]. By associating with a number of co-chaperones such as Hsp40, Hsp70 proteins achieve versatile functions in different cellular compartments [67]. 

## 3. Regulation of Chaperones

### 3.1. Transcriptional regulation: the heat shock response

Various stress conditions such as extreme temperature and fluctuation of oxygen supply can cause protein misfolding and other cellular damage. Cells respond by transcriptionally activating various protective chaperones, collectively known as the heat shock proteins. This heat shock response is regulated by a family of heat shock transcription factors (HSFs). Mammalian cells express multiple HSF genes (HSF1, HSF2, and HSF4), while *Drosophila*, *C. elegans*, and yeast express only HSF1 [68,69,70,71]. HSF1 is the key stress-responsive transcription factor in mammals [72], while HSF2 and HSF4 are essential for regulating developmental processes [73,74,75]. Upregulation of HSPs by HSF1 is triggered by a variety of acute and chronic stress conditions and disease states [69]. 

**Figure 1:**
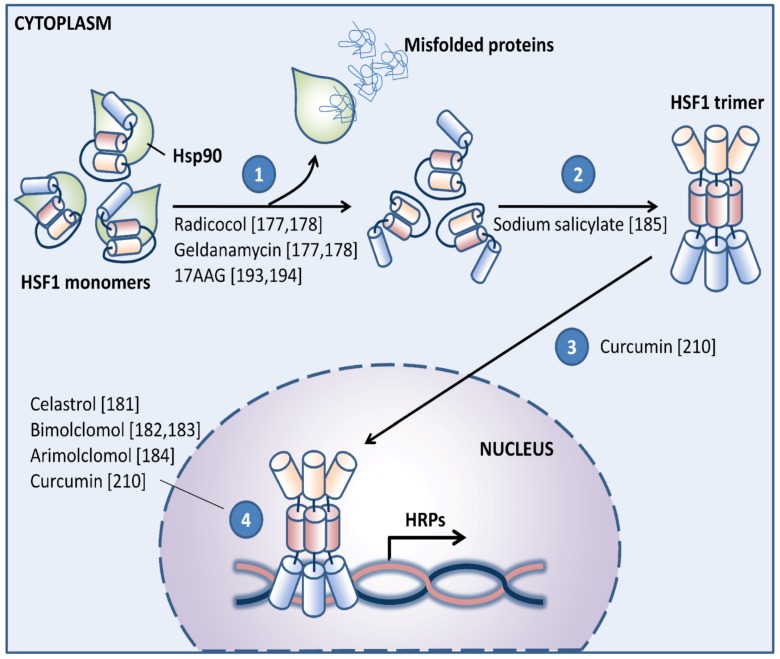
Induction of the heat shock response (HSR) and the expression of neuroprotective chaperones via pharmacological modulation of heat shock factor 1 (HSF1). Under normal physiological conditions, monomeric HSF1 is sequestered in the cell cytoplasm by constitutively expressed HSPs including Hsp90. Stress conditions activate the HSF1 mediated transcription of heat shock responsive protein (HRP) genes in a stepwise manner. Step 1, upon physiological stress (e.g., heat shock, proteotoxicity), HSPs dissociate from HSF1 to interact with misfolded proteins, allowing the release of HSF1 monomers. Step 2, free HSF1 monomers change conformation and trimerize. Step 3, HSF1 trimers are translocated into the nucleus. Step 4, upon further activation (e.g. phosphorylation, SUMOylation), HSF1 trimers bind to heat shock elements (HSEs) in the promoter/enhancer regions of HRP genes. Examples of compounds and small molecules that promote expression of HRPs by acting on these specific steps are shown. Radicocol, geldanamycin and 17AAG inactivate Hsp90 thereby derepressing monomeric HSF1 (Step 1). Sodium salicylate promote HSF1 trimerization (Step 2), Curcumin promotes HSF1 trimer transolcation (Step 3). Biolclomol and arimoclomol enhance the affinity of HSF1 for HSE binding (Step 4).

Under unstressed conditions, HSF1 is maintained in monomeric state by transient association with multi-chaperone complex of Hsp90, Hsp70 and Hsp40 [76,77,78]. Upon stress, a surge in chaperone demand from stress-induced protein misfolding sequesters HSF1-bound chaperones, and thus relieves HSF1 from its monomeric state [71]. Freed HSF1 monomers trimerize through an extended heptad repeat (HR-A/B) located between the DNA-binding domain and the transcription activation domain [79]. Trimerization is an essential step, as it exposes the DNA-binding domain of HSF1 and following nuclear localization, allows binding to heat shock elements (HSE) in respective candidate gene promoters, leading to upregulation of HSPs [69]. Binding of HSF1 to respective HSEs releases a pre-initiated paused RNA polymerase II complex upon recruitment of elongation factors including pTEFb [80]. HSPs are transcriptionally upregulated only when HSF trimers are bound to HSEs under prevailing stress signals [76]. The presence of stress is essential to the activation state of DNA-bound HSF1 via various post translational modifications such as phosphorylation [81,82,83] and sumoylation [84]. Reduced stress level will trigger a negative feedback loop where excess chaperones will sequester monomeric HSF1, thus attenuating HSF1-mediated transcription [68,69,71]. Therefore, a combination of post translational modifications and protein-protein interactions offers various levels of control on the stress-induced transcriptional activities of HSF1. A schematic diagram of the HSF1 activation is illustrated in Figure 1.

### 3.2. Post-transcriptional regulation: “minimal stress miRNAs”

MicroRNAs (miRNAs) play important roles in development, growth, and many other fundamental cellular processes [85,86,87]. Recently, miRNAs were also implicated in regulating cell stress response arising from diverse stress conditions such as heat and hypoxic stress [85,88,89]. Recent work by Wilmink *et al.* [90] uncovered several miRNAs (miR-125b, -222, -22, and let-7c) to be expressed in response to most stressor types, while some miRNAs are specific to thermal stress (miR-452, -382, and -378). More importantly, their work revealed a significant general down-regulation of miRNAs in response to stress. This is quite evident in stressed cells where demand for rapid protein translation interferes with the translation-repression role of miRNAs, allowing evolution of mechanisms that reduce miRNA synthesis during stress. Future work on analyzing the targets of these miRNAs will likely reveal an additional level of regulation to controlling stress protein expression. 

### 3.3. Post-translational modifications

The activity of some chaperones, like other proteins, has been shown to be regulated by different post-translational modifications. Reversible phosphorylation of the bacterial Hsp70 homolog DnaK and Hsp60 homolog GroEL was shown to affect their binding affinities to client proteins as well as ATPase activity [91,92,93]. Similarly, S-nitrosylation of Hsp90 was shown to inhibit its ATPase activity and prevent its regulation of a client protein, nitric oxide synthase [94], which is physiologically essential to initiate a negative feedback loop regulating the levels of nitric oxide. Other chaperones, such as Hsp33, are sensitive to cellular redox state, and thus provide an immediate response to oxidative stress. Under oxidative conditions, Hsp33 is activated by the formation disulfide bonds that release a zinc ion bound to the protein and ultimately lead to dimerization; under non-stressed conditions, this redox-sensitive chaperone is inactivated by the elimination of the disulfide bonds with reversal of these structural changes [95,96]. Such transient covalent modifications allow stress proteins to remain inert in normal conditions and to be rapidly switched on during stress. 

### 3.4. Allosteric regulation and co-chaperones

Many of the members of the HSP family, such as Hsp70, Hsp60, and Hsp90, are subject to allosteric regulation by nucleotides. Such regulation is common for many proteins whose activities are governed in response to the level of one or several small metabolites, such as ATP, which dictate the metabolic state of the cell. Many of the HSPs are known to exist in an ATP-bound and an ADP-bound state, allowing for differential binding affinities with client proteins [97,98,99,100]. Such interactions allow for continuous cyclic interactions between chaperones and their client proteins, providing a feedback regulation of chaperone function. Also, interactions with ATP and its subsequent hydrolysis into ATP provide “energy” for these chaperones to carry out their “mechanical” folding functions. 

In addition to the essential post-translational modifications and allosteric interactions, the activity of many chaperones would not be complete without interactions with other indispensible proteins called “co-chaperones”. The best studied co-chaperone is GroES, which helps GroEL (bacterial Hsp60) to function properly [10]. Extensive studies have shown that the key role of GroES is to enable GroEL to bind to the client protein in the central cavity and enable a conformational change and also help the release of the client protein. A large number of co-chaperones have also been indicated for Hsp90, helping regulate both its ATPase and client protein-binding activities [101].

## 4. Chaperones in Neurodegenerative Diseases

Many neurodegenerative diseases are considered “conformational” in nature as they are characterized by the accumulation of aberrantly folded protein species [102,103,104,105,106]. As neurons are a terminally differentiated, post-mitotic cell type, it has been suggested that they are especially susceptible to the cumulative effects of misfolded proteins as they are unable to reduce the load of toxic intermediates through consecutive rounds of mitosis [106]. Therefore, the capacity of neuronal chaperones to reduce misfolded proteins is essential for maintaining neuronal integrity. Moreover, most neurodegenerative diseases are characterized by typical misfolded protein aggregates, which have been shown to strongly colocalize with molecular chaperones (Table 1). A recent study comparing the expression levels of Hsc70 in different neuronal subtypes typically vulnerable in neurodegenerative diseases, including spinal motoneurons (vulnerable in amyotrophic lateral sclerosis (ALS)), neurons of the hippocampus/entorhinal cortices (vulnerable in Alzheimer’s disease (AD)), and tyrosine hydroxylase positive neurons of the substantia nigra (vulnerable in Parkinson’s disease (PD)), reported that the relative levels of Hsc70 expression was inversely correlated with the frequency of disease prevalence in the US population [107]. For example, hippocampal/entorhinal neurons express significantly less Hsc70 than both spinal moto- and substantia nigra neurons, while the frequency of AD is 4-fold higher than PD and 133-fold higher than ALS in the US population. This suggests that the vulnerability of particular subpopulations of neurons may be attributed to their variable pools of chaperones (e.g., reduced misfolded protein buffering capacity) [102]. Therefore, the induction of chaperone expression in neurons has become an active area of both basic science and translational research providing both a greater understanding of the underlying mechanisms of many neurodegenerative diseases as well as providing a new target for therapeutic design.

### 4.1. Parkinson’s disease (PD)

PD is the most common neurodegenerative movement disorder, affecting more than 0.1% of the population older than 40 years of age [108]. PD patients suffer from a motor disorder characterized by slowness of movement, rest tremors, rigidity, and disturbances in balance. PD is characterized by inclusion bodies called Lewy bodies formed by aberrant misfolding and aggregative proteins [109]. Chaperones, such as Hsp70 and Torsin A, a protein with homology to yeast Hsp104, are colocalized with α-synuclein (αSN) containing Lewy bodies [110]. Furthermore, Hsp70 was shown to inhibit αSN fibril formation by binding to prefibrillar oligomers and changing toxicity of αSN aggregates [111]. Mutations in DJ-1, a novel oncogene, are linked with familial PD and have been shown to cause oxidative stress and mitochondrial degeneration, leading to protein aggregation and neuronal cell death [112]. DJ-1 and its mutants are known to associate with Hsp70, CHIP (chaperone interacting protein) and mtHSP70/Grp75, which is enhanced with H_2_O_2_ treatment in cells [113]. Since stress chaperones play such a vital role in the pathogenesis of PD, it is important to note that the level of HSPs decreases significantly with aging, which leads to failure in cellular protein homeostasis, giving rise or contributing to such aging diseases [114].

It has been shown in *Drosophila* that overexpression of Hsp70 can prevent dopaminergic neuronal loss induced by αSN-overexpressing, and that reducing Hsp70 levels enhance αSN toxicity [115]. The likely mechanism of Hsp70-mediated protection in PD involves the recruitment of misfolded proteins as substrates for parkin E3 ubiquitin ligase and degradation of aberrant αSN [116]. In addition to its role in reducing proteotoxic stress, Hsp70 exerts anti-apoptotic activity by blocking the function of several key proapoptotic factors and also by activating the survival pathway in rotenone and MPTP induced sporadic PD models [117]. Increased expression and abnormal aggregation of small HSPs, such as αB-crystallin, is also a prominent feature of Lewy bodies and reactive astrocytes in various neurodegenerative diseases [118]. αB-crystallin overexpression reduces αSN fibrillization *in vitro* showing that it may revert fibril-αSN to an amorphous aggregation pathway, and help to reduce stable amyloid deposits to a more easily degradable form [119]. Hsp90, which exerts a negative effect on HSF1 regulation, was recently shown to be significantly increased in age-matched postmortem PD brains, along with a high level of insoluble αSN [120]. Increase of Hsp90 was also seen in αSN mutant transgenic mouse model of PD [120]. Therefore, increased Hsp90 level may inhibit the induction of stress chaperone and subsequently compromise the neuronal capacity to handle misfolded protein load.

### 4.2. Alzheimer’s disease (AD)

AD is the most common form of irreversible dementia and is characterized by a rapid progression from episodic memory problems to a decline in overall cognitive functions, impairing patients’ ability to carry out activities of daily living (ADL) and leading to death typically nine years following diagnosis [121]. The disease is characterized by the cortical presence of extracellular deposits of amyloid-β (Aβ), called senile plaques, and intraneuronal inculusions, called neurofibrillary tangles (NFTs), formed by accumulation of abnormal filaments of tau, both found predominately in the brain regions involved in memory and learning [122]. Postmortem expression studies of AD brain tissue has shown that a number of chaperones such as HSP27 and HSP70 are elevated in affected areas, which was partly a result of gliosis and stressed neurons [123,124].

Chaperones have been strongly implicated in AD pathology. Amyloid precursor protein (APP) interacts with the ER resident chaperone BiP/Grp78 (the ER isoform of Hsp70), during its normal processing in the ER-Golgi pathway [125]. Hence, increased Grp78 may help proper processing of APP therefore reduce amyloid production [125]. In addition, overexpression of cytosolic Hsp70 and Hsp90 has been shown to inhibit early stages of amyloid aggregation [126]. It has also been demonstrated that small HSPs such as Hsp22 and Hsp27 bind to fibrillar amyloid plaques and inhibit fibrillarisation [127]. Furthermore, overexpression of the small Hsp16.2 in *C elegans* is strongly protective against Aβ-induced toxicity [128].

The role of chaperones in AD has been studied extensively with respect to tau aggregation and fibrillization. Chaperones, including Hsp27, Hsp70 and CHIP were shown to colocalize with abnormal tau aggregates, and overexpression of these chaperones reduced hyperphosphorylation and increase misfolded tau degradation [129,130]. These chaperones were shown to preferentially bind to hyperphosphorylated tau as well as paired helical filamentous tau but not to non-phosphorylated tau. Moreover, increased levels of Hsp70 and Hsp90 promoted tau solubility and enhanced its binding to microtubules in various cellular models [131]. These studies have shown that chaperones are important for maintaining tau in its physiological form, bound to microtubules, and reduce its aggregation as a result of hyperphosphorylation.

### 4.3. PolyQ disease

PolyQ diseases are a family of dominant neurodegenerative diseases that are caused by proteins containing tandem polyglutamine repeats. At least nine polyQ related disorders are known to date, including spinal bulbar muscular atrophy (SBMA), Huntington’s disease (HD), dentatorubropallidoluysian atrophy (DRPLA), and six types of spinocerebellar ataxia (SCA1, 2, 3, 6, 7, and 17) [132]. PolyQ diseases are among the first characterized protein misfolding diseases in which neuroprotective properties of chaperones have been identified. Studies using different model systems have shown that overexpression of Hsp40 and Hsp70 can reduce polyQ toxicity and inclusion body formation [133,134]. Similarly, overexpression of Hsp27 was also shown to ameliorate polyQ toxicity, by reducing oxidative stress but without affecting inclusion body formation [135]. In yeast, overexpression of Ssa1 (Hsp70) or Ydj1 (an Hsp40 homolog) significantly reduces the formation of large, detergent-insoluble inclusion bodies and facilitates the accumulation of smaller aggregates [136]. Consistent with this, knocking down two of the Hsp70 isoforms in a *C elegans* model of polyQ disease accelerates disease progression [137]. Expression of a truncated form of ataxin 3 with a polyQ expansion (MJDtr-Q78) in *Drosophila* nervous system caused lethality that is rescued by co-expression of human Hsp70 [138]. Interestingly, Hsp70 expression did not affect inclusion body size, indicating that these aggregates might not be the toxic species. Furthermore, Hsc70 overexpression restores neuronal transport and significantly reduces cell death associated with polyQ pathogenesis [139]. Overexpression of Hsp70 in spinocerebellar ataxia 1 homolog (SCA1) transgenic mice (Ataxin 1 with 82 polyQ repeats) significantly improved the behavioral and pathological phenotypes, without causing any change in inclusion body formation [140].

### 4.4. Cerebral ischemia

Stroke, or focal reductions in cerebral blood flow (ischemia) as a result of blood vessel occlusion or hemorrhage [141], accounted for approximately one in every 18 deaths in 2006 and represents a significant cause of long term disability in the U.S. [142,143]. Ischemic injury to nervous tissue, including neurons, glia, and neurovasculature, is attributed to hypoxia and metabolic deficits that initiate a cascade of intracellular events, including ionic imbalance, oxidative/nitrosative stress, and excitotoxicity, ultimately resulting in necrotic or apoptotic cell death [141]. Recent studies have also observed that transient global and focal cerebral ischemia induces the accumulation of protein aggregates [144,145] and arrest of protein synthesis secondary to activation of the ER unfolded protein response (UPR) within cells soon after ischemic injury [146,147]. Proteotoxicity from ischemia induced protein aggregation may therefore further contribute to cell death following ischemic injury [148], especially in the periphery of flow compromised regions where milder ischemia is observed as a result of perfusion from nearby collateral vessels known as the penumbra [149]. In contrast to the zone immediate to the ischemic injury where cell death is more rapid and irreversible, cells within the penumbra die more slowly and may be rescued upon reperfusion [149]. Interestingly, upregulation of stress response proteins such as Hsp70 have been observed in a penumbra distribution [150,151], potentially an endogenous protective mechanism that distinguishes reversible ischemia in this region. Overexpression of Hsp70 has been demonstrated to reduce infarct size and protect both neurons and glia from cell death in several *in vivo* mammalian models of stroke [151,152,153,154,155,156] via mechanisms that directly inhibit apoptosis and injury secondary to immune activation [157] as well as by reducing ischemia induced protein aggregation [144,145,158]. Similar results have been obtained by overexpressing other heat shock response proteins such as Hsp27 [159,160] and the Hsp40 co-chaperone Hdj-2 [158,161]. Pharmacological induction of the constitutive ER chaperone BiP was also demonstrated to be neuroprotective by mitigating ischemia induced ER stress [162]. These observations and the fact that chaperones including Hsp70 can prevent both apoptotic and necrotic cell death has made heat shock response proteins ideal candidates in providing neuroprotection following cerebral ischemia [158].

**Table 1:** Association of molecular chaperones with neurodegenerative disorders.Examples of common neurodegenerative diseases with chaperones shown to colocalize with respective protein aggregates either in disease tissue or in experimental models. (Aβ= amyloid beta; APP= amyloid precursor protein; DJ1= Parkinson’s disease (autosomal recessive, early onset) 1; PINK1= phosphatase and tensin induced putative kinase 1; SOD1= superoxide dismutase 1).

Disease	Associated Genes	Pathology	Aggregate-Associated Chaperones	References
Alzheimer’s disease	APP, Presenilin1/2	Extracellular plaques of Aβ40 and Aβ42; Intracellular neurofibrillary tangles (NFTs)	Hsp72, Hsp28	[123, 124, 125, 126, 127]
			Hsp27, GRP78	
			Hsp27, Hsp90	
Parkinson’s disease	α-synuclein, Parkin, Pink1, DJ1	Intracellular Lewy Bodies	Hsp70, Hsp40, αβ-crystallin	[110, 111, 113, 115]
Familial ALS	SOD1	Intracellular inclusion bodies	Hsc70	[163]
Spinocerebellar ataxia (SCA1-3, 7)	Ataxins	Nuclear inclusions	Hsp40, Hsp70	[133, 134]

## 5. Diseases Caused by Mutations in Chaperones

With the completion of the human genome project, a lot of attention has been placed on linking mutations in genes with defined functions to disease susceptibility. Analysis of the open reading frames (ORFs) that contain mutations has revealed alterations in genes encoded for molecular chaperones that may be responsible for some complex human diseases.

Hsp60: The hereditary spastic paraplegia SPG13 is caused by a missense mutation of a conserved Valine (V72I) residue in the mitochondrial chaperonin Hsp60 [164]. The disease is characterized by weakness and poor coordination of lower limbs, resulting from severe degeneration of the distal ends of long axons in the spinal cord [165]. The relatively mild phenotype associated with the mutation is rather surprising, as Hsp60 is crucial for mitochondrial biogenesis and the missense mutation renders Hsp60 partially or completely nonfunctional. It is possible that compensatory mechanisms are in place to allow mitochondrial biogenesis without functional Hsp60 proteins.

hMKKS: Mutations in the cytosolic chaperonin hMKKS have been implicated in McKusick-Kaufman Syndrome (MKKS), characterized by malformations of the genital tract, polydactyly and congenital heart disease; and Bradet-Biedel type 6 Syndrome (BBS6), characterized by obesity, retinal dystrophy, renal malformations, learning and behavioral abnormalities and endocrinological dysfunction [166,167]. hMKKS is 40% similar to human TRiC protein, and is known to be expressed during development and in adults, but not much is known about its biochemical or structural properties. 

α-Crystallins: α-Crystallins are small HSPs, mutations in which have been linked with two diseases: R116C mutation in αA-crystallin is linked to a form of hereditary cataract [168]; and R120G substitution in αB-Crystallin results in a form of desmin-related myopathy (DRM) [169]. Although the precise mechanism by which mutant αA-Crystallin causes cataract is not known, it is predicted that the mutant chaperone fails to properly fold other major lens proteins. In the case of DRM, it is known that αB-Crystallin specifically interacts with desmin in muscle cells, possibly mediating its folding and/or assembly into intermediate filaments [170]. 

TBCE: The tubulin-specific chaperone E (cofactor E, TBCE) is an essential chaperone required for efficient folding of α-tubulin and its dimerization with β-tubulin. Mutations in TBCE lead to a reduction in the amount of available α-tubulin to be incorporated into microtubules, causing severe cytoskeletal defects such as aberrant polarity of microtubule network. Several severe diseases have been linked with mutation in this gene, including HRD (hypoparathyroidism, mental retardation and facial dysmorphism), Sanjad–Sakati syndrome, and autosomal recessive Kenny–Caffrey syndrome (AR-KCS) [171].

Sacsin: Sacsin was originally reported to consist of a single gigantic exon spanning 12.8 kb with an 11.5-kb ORF, with mutations linked to autosomal recessive spastic ataxia of Charlevoix–Saguenay (SACS) [172]. Recently, eight exons upstream from the original one have been found, and the new ORF has elongated to 13.7 kb. Sequence similarity revealed an ubiquitin domain and a DnaJ molecular chaperone homology domain preceding a HEPN (higher eukaryotes and prokaryotes nucleotide-binding) domain. The mutations R4325X and N4549D that cause SACS are present in the DnaJ domain and the HEPN domain, respectively [173]. The DnaJ heat shock domain is essential for interactions with chaperone Hsp70-like proteins and the HEPN domain is implicated in nucleotide binding [173]. This raises possibilities of Sacsin functioning either directly or indirectly in chaperone-mediated protein folding.

## 6. Pharmacological Modulators of Molecular Chaperones as a Therapeutic Approach

### 6.1. Small molecule regulators of the heat shock response

Most neurodegenerative diseases are “protein misfolding disorders” that could therapeutically benefit from active small molecules that regulate HSF1 or modulate chaperone activities that could possibly ameliorate the prevailing imbalance in protein homeostasis. 

Two of the first chemical inducers of the heat shock response were the protein synthesis inhibitor puromycin and the amino acid analog azetidine, both of which result in accumulation of prematurely-terminated new peptide chains or amino acid analog-containing misfolded proteins, igniting the stress response [174,175]. In following years, inhibitors of protein degradation, including proteasomal inhibitor MG132 and serine protease inhibitors such as DCIC and TLCK, were shown to induce HSF activation by inducing proteotoxic stress [176]. Potential inhibitors of Hsp90, including the antifungal agent radicocol and geldanamycin (a benzoquinone ansamycin), also activate HSF1 [177]. Both of these compounds have been shown to bind to the ATP-binding domain of Hsp90 and inhibit its activity, thereby de-repressing and activating HSF1 [178] (Figure 1). In mammalian cell culture and mouse brain slice culture models of Huntington’s disease, both drugs were shown to inhibit the aggregation of polyQ containing protein huntingtin and to be significantly neuroprotective [179,180]. The triterpenoid celastrol, a natural product from the *Celastraceae* plant family, is able to induce HSF1 with an EC50 in the micromolar range, making it highly suitable for therapeutics [181] (Figure 1). Other activators of HSF1 include the hydroxylamine derivative bimolclomol [182] (Figure 1), which was shown to be therapeutic in wound healing and ischemia [183], and arimoclomol which delayed disease progression in a mouse model of ALS [184]. 

Also important are compounds that are classified as co-inducers which partially activate components of the heat shock response. Belonging to this group are the non-steroidal anti-inflammatory drugs (NSAIDS), including sodium salicylate, which promotes trimerization of HSF1 without causing its activation [185]. Hence, co-inducers can only work in association with a stress signal for properly inducing the heat stress response. Long term use of NSAIDS for arthritis was shown to reduce the outcome of Alzheimer’s disease, further supporting a neuroprotective role of the heat shock response [186]. 

### 6.2. Chemical chaperones

Chemical chaperones are small molecular mass compounds that increase protein stability *in vitro.* These compounds include organic solvents such as dimethyl sulfoxide (DMSO) and osmolytes such as glycerol and trelahose [187,188]. Addition of DMSO and glycerol to scrapie-infected mouse neuroblastoma *in vitro* was able to effectively reduce the extent of the scrapie (abnormal) form of the prion protein and increase the normal cellular form of the protein [188]. In yeast, the synthesis of trehalose is responsive to stress and is important for maintaining protein stability during stress [189,190]. In fact, trehalose was shown to inhibit polyQ aggregation in R6/2 transgenic mouse model of Huntington’s disease [191]. In this study, feeding mice 2% trehalose in drinking water reduced disease pathology and improved motor deficits and lifespan. Consistent with this finding, deletion of the trehalose gene in yeast enhances α-synuclein toxicity [192].

### 6.3. Hsp90 inhibition: from cancer therapy to potential therapeutic for neurodegeneration

Hsp90 is an extremely important chaperone as it interacts with various kinases that have been implicated in malignant transformation, such as the ErbB2, Src, Abl or Met tyrosine kinases, or the Raf, Akt and cyclin-dependent serine kinases [56]. As such, targeting Hsp90 can ideally be therapeutic, as such inhibitors can act as multi-target drugs, which are more efficient than highly selective single-target drugs. The most widely used Hsp90 inhibitors are the macrocyclic antitumor agent geldanamycin and its less toxic analog, 17-allylamino-17-demethoxygeldanamycin (17AAG) [193]. In tumor cells, Hsp90 prevails in an activated large complex with various co-chaperones, in contrast to a latent, uncomplexed state in normal cells. Inhibitors such as 17AAG bind to the tumor-specific, complex form of Hsp90 with a 100-fold higher affinity than to the latent form in normal cells [194], which affords highly specific anti-tumor activity.

The primary focus on Hsp90 in neurodegenerative diseases has been limited to its inhibitory role in regulating the heat shock response [195]. More recently, new evidence suggests an additional role for Hsp90 in buffering some of the client proteins that might be directly responsible for disease progression [196,197]. In spinal and bulbar muscular atrophy (SBMA), an inherited motor neuron disease caused by the expansion of a polyglutamine tract within the androgen receptor (AR), the mutant AR is an Hsp90 client protein that forms a molecular complex with the chaperone [198]. In the same study, treatment of both cellular and mouse models of SBMA with 17AAG led to a preferential degradation and clearance of mutant AR compared to wild-type, indicating that mutant AR was maintained stably by complexing with Hsp90, and inhibiting Hsp90 promoted the clearance of mutant AR and led to amelioration of disease-associated motor impairments [198]. Another example of a potential therapeutic use of Hsp90 inhibitors is in tauopathies, or neurodegenerative disorders marked by abnormalities in tau phosphorylation leading to tau aggregation and neurodegeneration. In Alzheimer’s disease, tau hyperphosphorylation has been linked to aberrant activation of several kinases, such as cyclin-dependent protein kinase (cdk) 5 and glycogen synthase kinase (GSK) 3β [199]. Hsp90 has been shown to stabilize the P301L mutant tau protein and p35, a neuronal protein that may activate cdk5, while Hsp90 inhibition reduced the aberrant activity of these proteins and the level of aggregated tau, leaving normal tau largely unaffected [197].

### 6.4. Nutraceuticals

A majority of neurodegenerative diseases arise sporadically, revealing the importance of environmental factors that may contribute to disease progression or even dictate the outcome in some cases. Several epidemiological studies have indicated that dietary habits and antioxidants from diet can influence the incidence of neurodegenerative disorders such as Alzheimer's and Parkinson's diseases [200,201]. For example, results from the Personnes Agees Quid study showed that people consuming three to four glasses of wine per day had an 80% decreased incidence of dementia and Alzheimer's disease three years later, compared to people who drank less or not at all [202]. Consumption of tea was shown to reduce the risk of Parkinson's disease [203]. Moreover, in a separate study on a population of 65 years of age and above, there was a striking inverse relationship between flavonoid intake (fruits, vegetables, wine and tea) and the risk of dementia [204]. Similarly, a positive correlation has been shown between the consumption of the *Ginkgo biloba* extract EGb 761 and improved cognitive performance in Alzheimer's patients [205,206]. These clinical and epidemiological results indicate a protective effect of flavonoids and polyphenols in neurodegenerative diseases. 

Polyphenols are natural substances present in plants, fruits, vegetables, and processed foods including olive oil, red wine, and tea. Flavonoids represent the largest group of polyphenols. There is increasing evidence that consumption of foods or beverages rich in polyphenols is beneficial and can increase the antioxidant levels in serum protecting against oxidative-induced damage [207]. Apart from being great scavengers of free radicals, some polyphenols may directly stimulate the stress response and increase cellular chaperone levels. Resveratrol, a polyphenol from red wine, is a known inducer of the heat shock response, shown to upregulate chaperones like Hsp70 in pre-treated human cell lines, when compared to moderate heat shock stress [208].

Curcumin, a non-flavonoidic polyphenol derived from the yellow curry spice, displays anti-inflammatory and antioxidant activities. Studies indicate this molecule, widely used by Indian and other South and East Asian populations, to be responsible for the significantly lower prevalence of Alzheimer's disease in India compared to the United States [209]. Curcumin is cytoprotective and induces nuclear translocation of HSF1 and its activation through the extracellular regulated kinase (ERK)/mitogen activated protein (MAP) and c-jun N-terminal kinase (JNK) pathways [210] (Figure 1).

## 7. Conclusions

Neurodegeneration, or the broad or selective loss of neuronal populations, is a common underlying feature of many inherited and acquired diseases, and injury of the central (CNS) and peripheral nervous systems (PNS). Their long life and often enormous size make neurons highly susceptible to all types of intracellular or extracellular insults. Cellular stress response is an evolutionarily conserved defense mechanism to protect cells against insults. Chaperones are the major players in the stress response. They are proteins often conserved over distant phyla. Neurons are endowed with the full repertoire of chaperones; however their importance in neuroprotection only became evident recently. The neurons’ high demand on maintenance and self-protection predicts the indispensable role of chaperones in neuronal maintenance and protection. A failure of such a maintenance system, or toxic stress at levels surpassing the maintenance capacity, would lead to neurodegeneration. Indeed, several mutations in chaperones have been linked to specific neurodegenerative diseases. It is also expected that compromised activity or function of chaperones would increase the susceptibility of an organism to neurodegeneration. Conversely, enhancing the maintenance and stress response capacity will increase neurons’ tolerance to internal or external insults. Recent findings that increased neuronal levels of specific chaperones have neuroprotective capacity in several neurodegenerative disease models further underscore the importance of understanding the regulatory mechanisms of chaperones. As exemplified by the HSF1 pathway, where the neuroprotective properties of several compounds and small molecules have only been revealed after the elucidation of the regulatory mechanisms of HSF1 (Figure 1), future studies on dissecting the intricate regulatory network in neuronal chaperones and stress response will be fruitful and will not only contribute to the understanding of neuroprotection but also serve as a stepping stone towards therapeutic design for neurodegenerative diseases.
